# The Prevalence and Antibiotics Susceptibility Patterns of *Corynebacterium minutissimum* Isolates from Skin Lesions of Patients with Suspected Erythrasma from Tabriz, Iran

**DOI:** 10.1155/2022/4016173

**Published:** 2022-11-30

**Authors:** Seyedeh Zahra Salemi, Mohammad Yousef Memar, Hossein Samadi Kafil, Javid Sadeghi, Hamideh Herizchi Ghadim, Hamideh Azimi Alamdari, Javad Nezhadi, Reza Ghotaslou

**Affiliations:** ^1^Infectious and Tropical Diseases Research Center, Tabriz University of Medical Sciences, Tabriz, Iran; ^2^Department of Bacteriology and Virology, Faculty of Medicine, Tabriz University of Medical Sciences, Tabriz, Iran; ^3^Tabriz University of Medical Sciences, Tabriz, Iran; ^4^Drug Applied Research Center, Tabriz University of Medical Sciences, Tabriz, Iran; ^5^Department of Dermatology, School of Medicine, Tabriz University of Medical Sciences, Tabriz, Iran; ^6^Sina Medical Research and Training Hospital, Tabriz, Iran

## Abstract

Erythrasma is a chronic infection of the skin that appears in the body folds as flat copper spots. The causative agent of this infection is *Corynebacterium minutissimum* (*C. minutissimum*). Erythrasma can be treated with antiseptics or topical antibiotics. The study aimed to investigate the antibiotics susceptibility patterns, and the presence of the erythromycin resistance gene (*ermX* and *mefA*) in *C. minutissimum* isolates in skin lesions with suspected erythrasma. From July 2020 to May 2022, 278 skin scrub specimens were collected from patients admitted to the hospital of Tabriz University of Medical Sciences. Specimens were incubated on the blood agar plates and isolates were identified by microbiological laboratory methods. The antibiotic susceptibility patterns were determined by the disk diffusion method and resistance genes of *ermX* and *mefA* were detected by the PCR method. Out of 278 specimens, 41 *C. minutissimum* isolates (14.74%) were recovered. The highest frequency of resistance was observed to a penicillin (75.6%) followed by erythromycin and clarithromycin (39.02%), clindamycin (30.05%), tetracycline (24.2%), and gentamicin and neomycin (19.5%). The frequencies of *ermX* and *mefA* genes were 75% and 12.5%, respectively. Resistance to antimicrobial drugs was common and worrying. Resistance to erythromycin in *C. minutissimum* is mainly related to the *ermX* gene.

## 1. Introduction

Erythrasma is a chronic infection of the skin's outer layers that are often found in the folds of the body such as the navel, groin, under the breasts, and underarms. This infection is associated with mild inflammation without vesicle secretion, but it is accompanied by mild and often asymptomatic scaling. It is less common in the armpit but is more common in the foot and then in the groin, which is the most common site of skin infection [1]. Erythrasma is a worldwide disease and its prevalence has been reported from 4 to 15% in the general population and up to 40% in athletes. Erythrasma is often reported in hot and humid areas and often occurs in adults and is less common in children. Worldwide, it is more common in males than females. The incidence of erythrasma may increase with age [2–4]. In the elderly, it accounts for 17.6% of bacterial skin infections and 44% of foot infections in patients with diabetes. Erythrasma has been reported from Iran as the second most common infection (35%) among patients with superficial and cutaneous intertriginous infections [5]. The rate of erythrasma has been reported at 46.7% among patients with clinically suspected tinea pedis from Turkey [6]. Predisposing factors that increase the risk of erythrasma include obesity, diabetes, hot and humid environments, skin disorders, and excessive sweating. Lack of personal hygiene and increased humidity can exaggerate the symptoms of the disease [7, 8]. Skin lesions in erythrasma may often be confused with fungal infections.

The causative agent of erythrasma is a bacterium named *Corynebacterium minutissimum* (*C. minutissimum*) that is classified taxonomy as phylum Actinobacteria, class Actinomycetia, order Corynebacteriales, and belongs to the family Corynebacteriaceae. *C. minutissimum* is gram-positive, nonacid fast, catalase-positive, nonspore-forming, urease-negative, nonmotile, gelatinase-negative, and facultative anaerobic bacillus. *C. minutissimu*m is the normal microbiota of the skin and belongs to the group of diphtheroid bacteria [9–11].

To manage erythrasma, there are several topical and oral treatments including clindamycin or erythromycin, and clarithromycin [1]. Antibiotic resistance has risen to warn levels around the world, and new mechanisms of resistance are emerging and expanding globally, threatening our ability to treat common infectious diseases such as erythrasma [12]. Therefore, it is necessary to reevaluate the available therapeutic drugs for the treatment of drug-resistant infections caused by *C. minutissimum*. The aim of this study was to evaluate the frequency of *C. minutissimum* from the skin lesions with suspected erythrasma, characterization of antibiotic susceptibility pattern, and the presence of the erythromycin resistance genes (*ermX* and *mefA*) in isolates from Tabriz, Iran.

## 2. Material and Methods

### 2.1. Patients and Specimens

In this cross-sectional study, which was performed for two years from July 2020 to May 2022, 278 skin specimens were collected from patients in ambulatory care (outpatients) admitted to Imam Reza and Sina Hospitals (two large and teaching hospitals in Tabriz, Iran). The patients were visited by dermatologists. The inclusion criteria were patients with suspected erythrasma (symptoms include itchy, axillary, and groin lesions, circumscribed, erythematous, brownish, scaly plaques affecting the armpits and the groin) and exclusion criteria were topical usage of antibiotics in the past two weeks. Skin scrape specimens were taken from the creased areas of the body, such as the armpits, groin, toes, and other areas of the body, using a scalpel and the skin was scraped.

### 2.2. Microbiological Procedure

Specimens were transferred to the microbiology laboratory. The specimens were homogenized in normal saline and were inoculated on the sheep blood agar (SBA) plate, and incubated at 37°C for 48 h under CO_2_ enriched atmosphere. To identify the *C. minutissimum,* gram staining and biochemical tests including catalase, oxidase, motility, hydrolysis of hippurate, methyl red, acid production from glucose, maltose, and saccharose, and urease tests were used [13].

### 2.3. Antimicrobial Susceptibility Testing

To determine antibiotic susceptibility, the disk diffusion method was performed according to the European Committee on Antimicrobial Susceptibility Testing (EUCAST) [14]. For this purpose, a bacterial suspension with a turbidity of 0.5 McFarland was prepared and then inoculated on the Mueller-Hinton agar +5% defibrinated horse blood and 20 mg/L *β*-NAD (MH-F). The disks of antibiotics including tetracycline (30 *µ*g), gentamicin (10 *µ*g), erythromycin (5 *µ*g), penicillin (10 *µ*g), neomycin (120 *µ*g), clarithromycin (15 *µ*g), and clindamycin (2 *µ*g) were used. All antibiotic disks were provided by Mast Ltd, England. The plates were incubated at 37°C for 24 h. The results for tetracycline, erythromycin, clindamycin, penicillin, and aminoglycosides were interpreted using the EUCAST breakpoints, and results for clarithromycin were interpreted using the CLSI breakpoints suggested for *Streptococcus pneumoniae* (*S. pneumoniae*) [14, 15]. The results of antibiotic susceptibility testing were validated using the control strain of *S. pneumoniae* ATCC 49619 [14].

### 2.4. Polymerase Chain Reaction (PCR)

DNA was extracted from bacterial colonies growing on the BHI agar using the boiling method [16]. The specific amplification primers used for PCR are listed in Table 1 [17, 18]. PCR was performed in the reactions with a final volume of 20 *μ*L including 5 *μ*L of master mix 1X (SinaClon Co., Iran) containing Taq DNA polymerase, MgCl_2,_ and dNTPs. Amplification reactions were conducted in an Eppendorf thermocycler with an initial denaturation (for 10 min at 92°C), followed by 34 cycles of denaturation (for 30 s at 92°C), annealing (for 45 s at 56°C), and then extension (for 40 s at 72°C) with a final extension for 10 min at 72°C. Strains of *C. minutissimum* that were previously confirmed for the presence of *ermX* and *mefA* genes were used as positive controls and strains that were negative for the presence of genes were used as negative controls in all stages of PCR. The PCR products were resolved using the gel electrophoresis on 1% agarose gels in 0.5× TBE buffer. The gels were stained with DNA-safe stain and were visualized under ultraviolet light. The sizes of the PCR products were determined by comparison with a molecular size marker (100 bp DNA ladder).

### 2.5. Statistical Analysis

The sample size was determined based on an expected frequency (a priori estimate of frequency according to a pilot study result), an accepted error of 4% (required precision of the estimate), and a 95% level of confidence. The results were analyzed by SPSS.ver 26 software. Fisher's exact test or Chi-square was applied to evaluate the association between the presence of *ermX* and *mefA* genes with resistance to erythromycin. *P* values ≤0.05 were considered statistically significant.

## 3. Results

**Figure 1:**
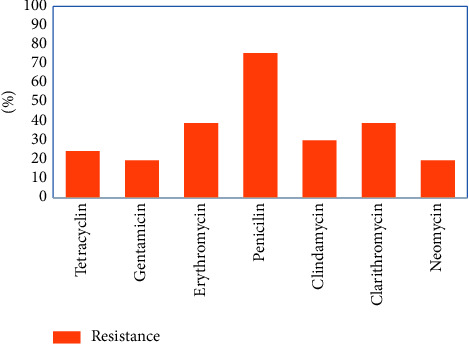
Antibiotic resistance patterns of *C. minutissimum* in the present study.

The skin lesions with suspected erythrasma were collected from 278 cases including 103 females and 175 males. The age of patients was from 15 to 72 years. The prevalence of *C. minutissimum* infection among skin lesions with suspected erythrasma was 41 (14.7%). Twenty-nine (70.7%) positive samples were males and twelve (29.2%) were females. The average age of patients with erythrasma was 45.5 years. The highest frequency of resistance was observed by penicillin (75.6%) followed by erythromycin and clarithromycin (39.2%), clindamycin (30.0%), tetracycline (24.2%), and gentamicin and neomycin (19.5%) (Figure 1).

**Figure 2:**
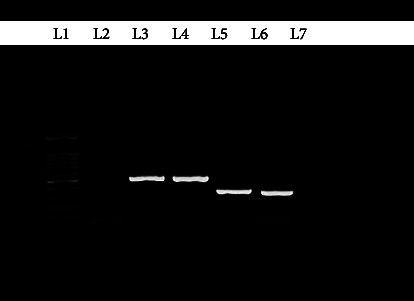
Gel electrophoresis of the PCR products. L-1: 100-bp ladder; L-2: negative control of *ermX* and *mefA* genes; L-3: positive control for *C. mintissimum* containing the *ermX*; L-4: *ermX* gene; L-5: positive control of *mefA*; L-6: *mefA*.

Among erythromycin-resistant isolates, *ermX* and *mefA* genes were observed in 75% and 12.5% of isolates, respectively (Figure 2). There was no significant relationship between antibiotic resistance and sex and age. There was a significant relationship between antibiotic resistance to erythromycin and the presence of the *ermX* gene (*P* < 0.05).

## 4. Discussion

In the current study, the prevalence of *C. minutissimum* isolates from skin lesions of patients with suspected erythrasma, the patterns of antibiotic resistance, and the frequency of erythromycin resistance genes (*ermX* and *mefA*) were investigated. The prevalence of *C. minutissimum* isolates from skin lesions of patients with suspected erythrasma was 14.7%, which was similar to a study in Iraq (17%) and a study in Turkey (15%) [19, 20]. The prevalence of infections due to *C. minutissimum* in our study was lower than in another study from Turkey (46.7%) [6], Mexico (32.8%) [21], and Bulgaria (40%) [2]. In a study by Janeczek et al. [22], erythrasma was detected in 56.6% of patients with psoriasis. The reason for the high prevalence of erythrasma may be due to humidity, hot weather, age, sex, diabetes mellitus, living in institutions, socioeconomic states, public baths (erythrasma-causing bacteria can survive for months and years in baths and pools), wearing shoes for long periods, and excessive sweating, which contributes to the growth of bacteria. Walking barefoot facilitates the transmission of infection. However, different prevalences in various countries can be partly due to the different clinical and laboratory diagnostic criteria in the identification of erythrasma-causingmicro-organisms. Although direct microscopic assay is applicable for providing preidentification data, the culture method enhances the ratio of identification. It was reported that several factors may affect the sensitivity of direct microscopic assay and the culture base methods including the methods of specimen collection, transfer of specimens to the laboratory, the quality level of the laboratory, and the previous antibiotic therapy of patients [23].

The CLSI has published minimum inhibitory concentration (MIC) breakpoints for Corynebacterium spp. However, the breakpoints for the disk diffusion method have not been established by CLSI. Due to the usefulness of disk diffusion in daily practice, some studies have been performed to compare disk diffusion with MIC- determining methods for antibiotics susceptibility testing of Corynebacterium spp [12].

There is evidence to indicate the increasing resistance rate of *C. minutissimum* to different antimicrobial agents. In the current survey, the resistance rate of penicillin was 75.6%. This result is similar to a previous report in Brazil [24]. However, the resistance frequency to penicillin in some previous studies was higher than our findings, such as Turkey (95%) [25] and Canada (96%) [26]. Due to the high frequency of resistance to penicillin, this drug is not an appropriate option for the therapy of infections caused by *C. minutissimum*. The mechanism of resistance to *β*-lactam agents in Corynebacterium spp. is not clearly described; however, it is likely due to reduced cell wall permeability or affinity for penicillin [12].

Most recent studies show an alarming frequency of antibiotic resistance to macrolides among *C. minutissimum*. In our study, the erythromycin resistance rate was 39.2%, which is lower than the findings of studies from Canada (81.9%) [27], Brazil (45%) [24], and Turkey (95%) [25]. In our work, erythromycin was less active than tetracycline, clindamycin, and gentamicin.

The clindamycin resistance rate was observed in 30.05% of isolates which was lower than the findings of a study from Egypt (98.7%) [28]. The tetracycline resistance rate was observed in 24% of isolates, which was similar to the study in Brazil (22%) [24], lower than in Switzerland (42%) [25], and higher than in Canada (7.9%) [26]. The gentamicin resistance rate (19.51%) was lower than the reported findings from Brazil (45%) [24], Canada (96.2%) [26], and Egypt (50%) [28]. The clarithromycin resistance rate in Brazil was 56% [24], which was higher than the resistance rate (39.02%) in our study. Differences in antibiotic resistance patterns in various studies may be due to different patterns of antibiotic usage. As you know, the different socioeconomic status, bacteria isolated from outpatients or inpatients, the efficacy of infection control practices, healthcare facilities, culture, and behavioral factors may also have a major impact on the amount of antibiotic administration and the prevalence of resistance to different pathogens in the world.

PCR has been used to detect the genes encoding erythromycin ribosome methylases (rRNAs) that are associated with erythromycin resistance. Subsequently, the resistance mechanisms have been investigated for various species, including *mef* (efflux pump) genes and *erm* genes family. *ermX* and *mefA* are the most common resistance genes reported from corynebacterium isolates [29]. The *erm* gene, thought to be transported on plasmid transposons, adds one or two methyl groups to a single adenine in the 23S rRNA, creating a high level of resistance to macrolides, lincosamides, and streptogramin B (MLS phenotype) [29]. In our study, the *ermX* and *mefA* were detected in 75% and 12.5% of isolates, respectively. In a study from Spain, all erythromycin-resistant isolates had the *ermX* gene [30], another study from Spain showed that 91% of erythromycin-resistant strains had *ermX* gene [31]. A study from Canada reported that the frequency of *ermX* gene in resistant strains was 97%. The different rates of resistance genes in the previous studies could be due to the differences in geographical areas and the amount and kind of antibiotic usage.

We acknowledge some limitations of the present study. First, the mechanism of resistance to antimicrobial agents other than erythromycin was not studied, which may be helpful for epidemiological surveillance and control programs of infections. In addition, we were not determined the susceptibility patterns to some effective antimicrobial agents against gram-positive bacteria, such as vancomycin, linezolid, and azithromycin, which can be considered empirical alternative therapeutic options.

## 5. Conclusion

The rate of *C. minutissimum* isolated from skin lesions with suspected erythrasma is relatively moderate. The majority of the study population is adult males, which indicates that males are more at risk for erythrasma. Penicillin is not a suitable choice for the therapy of infections caused by *C. minutissimum.* Resistance to erythromycin, the drug of choice for the treatment of erythrasma, is almost high, indicating the proper use of antibiotics and infection control strategies to prevent the spread of antibiotic resistance in the community environment.

## Figures and Tables

**Table 1:** Primers for amplification of genes.

Genes	Designation	Sequence (5′–3′)	Product size (bp)	Reference
*mefA*	*mefA*1	AGTATCATTAATCACTAGTGC	348	[17]
	*mefA*2	TTCTTCTGGTACTAAAAGTGG		

*ermX*	*ermX* up	AACCATGATTGTGTTTCTGAACG	566	[18]
	*ermX* down	ACCAGGAAGCGGTGCCCT		

## Data Availability

The data that support the findings of this study are available and included within the article.
